# *Potentilla paradoxa* Nutt. Ethanol Extract Exhibits Anti-Inflammatory Effects by Suppression of the Src/NF-κB Signaling Pathway

**DOI:** 10.3390/plants11131750

**Published:** 2022-06-30

**Authors:** Ji Won Kim, Ki Woong Kwon, Mi-Yeon Kim, Jae Youl Cho

**Affiliations:** 1 Department of Integrative Biotechnology, Sungkyunkwan University, Suwon 16419, Korea; lauryun@naver.com (J.W.K.); nexus0322@naver.com (K.W.K.); 2 School of Systems Biomedical Science, Soongsil University, Seoul 06978, Korea; 3 Research Institute of Biomolecule Control and Biomedical Institute for Convergence at SKKU (BICS), Sungkyunkwan University, Suwon 16419, Korea

**Keywords:** *Potentilla paradoxa* Nutt., anti-inflammatory effect, NF-κB signaling pathway, Src, gastritis

## Abstract

Inflammation is an immune response that protects against harmful stimuli. However, severe inflammation can cause many diseases, such as diabetes, cancer, and arthritis. In this study, we examined the anti-inflammatory efficacy and mechanism of *Potentilla paradoxa* Nutt. ethanol extract (Pp-EE) as a new strategy for controlling the inflammatory response. Cellular activities and the molecular target of Pp-EE were identified in RAW264.7 cells and HEK293T cells. The effect of Pp-EE was analyzed using the Griess assay, the luciferase assay, reverse transcription-polymerase chain reaction, and Western blotting. To evaluate the in vivo effects, an HCl/EtOH-induced gastritis mouse model was used. NO production and pro-inflammatory gene (*iNOS*, *COX-2*, and *TNF-α*) mRNA levels were decreased by Pp-EE in a concentration-dependent manner without showing cytotoxicity. The activation of the transcription factor, particularly NF-κB, was effectively suppressed by Pp-EE. It was also found that Pp-EE directly inhibits the activation of Src in lipopolysaccharide (LPS)-treated RAW264.7 cells and in Src-overexpressed HEK293 cells by Western blotting analysis and cellular thermal shift assay. Experiments in the gastritis mouse model indicated that Pp-EE suppresses HCl/EtOH-induced gastric lesions, the expression levels of *COX-2*, *IL-6*, and *TNF-α*, and the phosphorylation of p65, p50, and Src. Taken together, these results suggest that Pp-EE can be applied as an anti-inflammatory remedy with a Src/NF-κB inhibitory property.

## 1. Introduction

Inflammation is a vital part of the body’s innate defense mechanism that protects the body from external stimuli that are considered a threat to our body [1]. In response to a wound or infection, various kinds of immune cells (neutrophils, monocytes, macrophages, and eosinophils) participate in this mechanism by secreting various substances that include growth factors, eicosanoids, complements, and peptide inflammatory cytokines to remove the injurious stimuli and then initiate the healing process [2,3,4]. Therefore, inflammation is an essential defense mechanism for health. Inflammation can be divided into acute and chronic inflammation. Acute inflammation is described as the activation of temporary and restrictive inflammatory responses that occur when threats exist and then disappear as they are resolved [5]; it is considered a healthy inflammation. However, chronic inflammation acts as another threat to our bodies [6]. Chronic inflammatory conditions are chronic low levels of long-term inflammation in our body, regardless of infection. This can break the immune tolerance, so it becomes hard for our body to function properly at the cell level, affecting normal systems and organs. Therefore, the impaired system makes our bodies more vulnerable to infections or tumors and can reduce the responsiveness to vaccines [7,8,9,10]. Additionally, it becomes hard to distinguish between self or non-self, inducing autoimmune diseases such as rheumatoid arthritis; if septic shock occurs due to excessively produced inflammatory substances, it may lead to death.

The immune system of our body, to which the inflammation belongs, is innate immunity. Innate immunity is a non-specific first line of defense that is inherent from birth and begins with the release of cytokines and granules when cells such as neutrophils, macrophages, and mast cells become activated early in infection. Toll-like receptors (TLRs) play the most crucial role in the onset of these immune responses. TLRs are located on cell surfaces or the intracellular compartments of cells. TLRs are classified into several types according to their location and recognition, and each receptor plays a crucial role by recognizing specific pathogen-associated molecular patterns (PAMPs), such as lipids, nucleic acids, or others. When pattern recognition receptors (PRRs) such as TLRs recognize PAMPs, intracellular signaling takes place and then triggers downstream signaling cascades and the production of pro-inflammatory cytokines and chemokines [11,12]. For example, TLR4 promotes immune response by recognizing lipopolysaccharide (LPS), one of the endotoxins released from Gram-negative bacteria. In the cytosolic part of TLRs, the key signaling domain, the Toll-interleukin receptor (TIR) domain, initiates these signaling cascades while interacting with adaptor proteins such as myeloid differentiation factor 88 (MyD88) and TIR domain-containing adaptor-inducing IFN-β (TRIF). Adaptor proteins have a binding site that can bind specifically to various molecules, thereby leading to a series of signaling cascades by binding with molecules [13,14]. When Src and Syk, the protein kinases, are phosphorylated by the adaptor protein and are activated, molecules such as phosphoinositide 3-kinase (PI3K), protein kinase B (AKT), and IκB kinase (IKK) are activated in turn to transmit signals [15]. As a result, transcription factors such as nuclear factor kappa-light-chain-enhancers of activated B cells (NF-κBs) or activator protein-1 (AP-1) are translocated to the nucleus. Both transcription factors play a prominent role in the development of inflammatory reactions by promoting the expression and secretion of chemokines and cytokines that attract and activate immune cells [16]. As a key modulator of inflammation, various cytokines interact with cells in a network, allowing the immune system to function.

The Src kinase (Src) is a key factor in innate immunity as it regulates the development and functional responses, proliferation, and survival of immune cells [17,18]. In particular, Src, found to be involved in the signaling of all TLRs, increases the nuclear translocation of subunits of transcription factors such as c-Jun and p65 [19] and regulates morphological changes and phagocytosis in macrophages through TLR4 receptors [20]. Additionally, Src is activated by inflammatory cytokines, so it acts as a link for chronic inflammation to be associated with cancer [21,22]; it is important to regulate the activity of Src at a non-excessive level to maintain our health.

*Potentilla paradoxa* Nutt. grows in a wide range of northern hemispheres, including Asia, Europe, North America, and North Africa [23], and its roots are known to act as hemostasis, fever, and tonic agents. This plant is used for the treatment of astringent and febrifuge in India, pieces of the root are held in the mouth to relieve toothache, and the juice of the root is used in the treatment of indigestion in Nepal [24]. It has also recently been revealed that ethanol extracts of the plant display anti-oxidative, anti-melanogenic, would-healing, and skin-moisturizing activities [25]. Previously, there have been studies showing that various plants belonging to *Potentilla* have anti-inflammatory effects, and there have been studies on the anti-inflammatory effect of *P. paradoxa* Nutt. ethanol extract (Pp-EE); however, in those papers, specifically related cytokines and protein expression mechanisms were not identified [26,27]. Therefore, we aim to identify the mechanism of action at the unknown molecular level. In order to achieve this goal, we performed the following experiment using activated macrophages by LPS/TLR4 stimulation and HCl/EtOH-induced gastritis mouse model.

## 2. Results

### 2.1. Pp-EE Suppressed the NO Production Level and Phytochemical Components in Pp-EE

**Figure 1:**
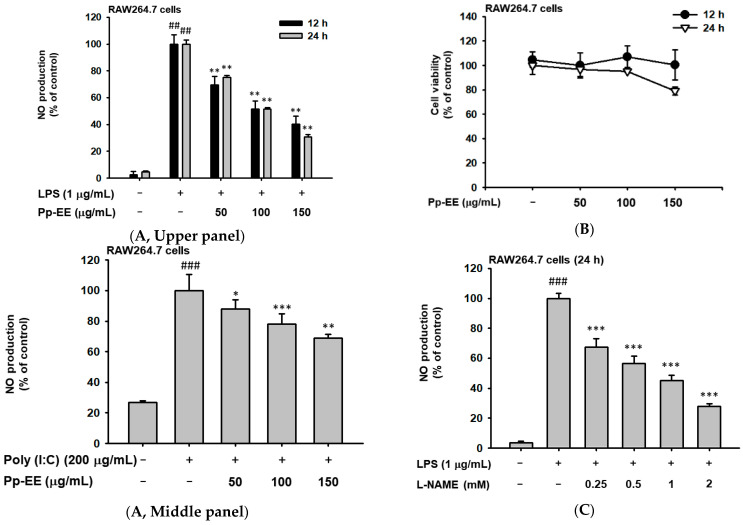

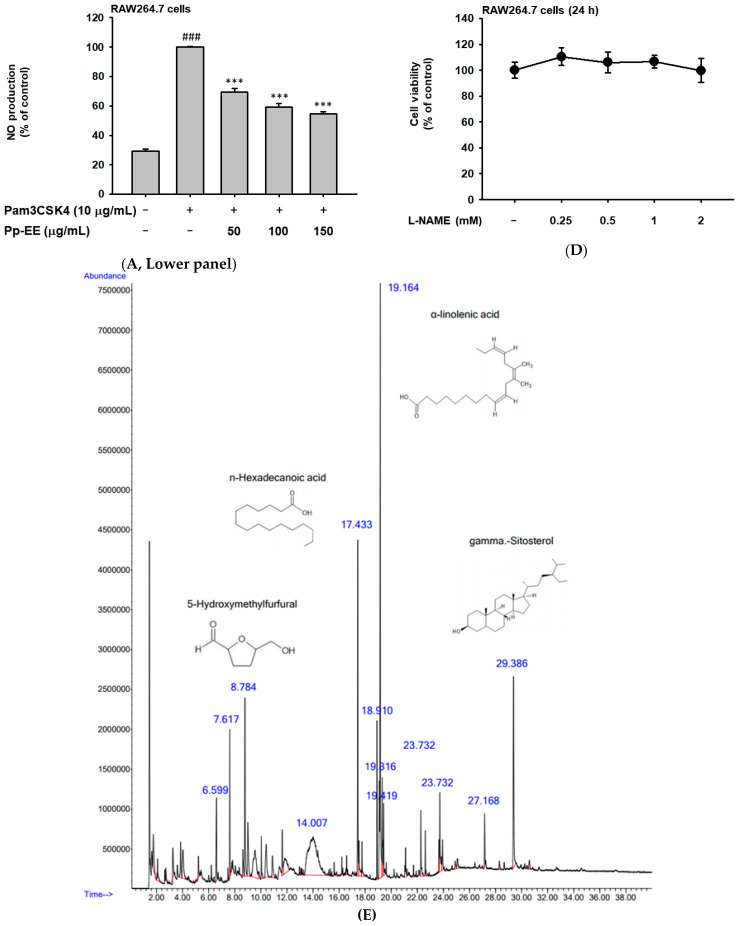
Effect of *Potentilla paradoxa* Nutt. ethanol extract (Pp-EE) on NO production and cell viability. (**A**) NO production from the supernatants of RAW264.7 cells pretreated with various concentrations of Pp-EE (50–150 μg/mL) for 30 min and treated with respective inflammatory substances (lipopolysaccharide (LPS) (1 μg/mL) (upper panel), polyinosinic:polycytidylic acid (Poly (I:C)) (200 μg/mL) (middle panel), or Pam3CysSerLys4 (Pam3CSK4) (10 μg/mL) (lower panel)) for 24 h. The NO production level was measured through a Griess assay. (**B**) Cytotoxicity of Pp-EE in RAW264.7 cells treated for 12 h and 24 h. The cell survival rate was measured with an MTT assay. (**C**,**D**) Treatment of N(G)-nitro-L-arginine methyl ester (L-NAME) (0–2 mM) in RAW264.7 cells for 24 h for efficacy comparison. Changes in NO production and effects on cell visibility were measured. (**E**) The analysis of phytochemicals in Pp-EE by GC–MS. The name and molecular structure of high-content phytochemicals are displayed. ###: *p* < 0.001 and ##: *p* < 0.01 compared to the normal group, *: *p* < 0.05, **: *p* < 0.01, and ***: *p* < 0.001 compared to the control group. −: no treatment and +: treatment.

To test the anti-inflammatory effect of Pp-EE, we firstly confirmed the change in the production amount of nitric oxide. Nitric oxide (NO) is a signal transmission molecule that plays an important role in the occurrence of inflammation, and through this, it is possible to confirm whether inflammation occurs [28]. We treated RAW264.7 cells with LPS (ligand of TLR4), polyinosinic:polycytidylic acid (Poly (I:C), a ligand of TLR3), and Pam3CysSerLys4 (Pam3CSK4, a ligand of TLR1/2) to induce an inflammatory response through TLRs. In all experiments, as the concentration of Pp-EE increased, the production of NO was significantly decreased (Figure 1A). Cell viability assay was also performed to evaluate the cytotoxicity of Pp-EE in RAW264.7 cells, and the cytotoxicity was not exhibited when the samples were treated for 12 and 24 h (Figure 1B). Through this, it was confirmed that the reduction in NO production was not a result of cytotoxicity. Additionally, to compare these anti-inflammatory effects, we check the inhibitory effect of N(G)-nitro-L-arginine methyl ester (*L*-NAME), a well-known NO inhibitor, as a positive control. As a result, it was confirmed that the tendency was similar to that shown in Pp-EE, and the effect of Pp-EE was comparable to that of the drug used as a positive control group (Figure 1C,D).

Next, we conducted GC–MS analysis to manifest the component that has the anti-inflammatory effects of Pp-EE. We analyzed the phytochemical components, and active ingredients such as α-linolenic acid, *n*-hexadecoic acid, and 5-hydroxymethylfural were observed in the order of high component content for Pp-EE (Figure 1E). α-Linolenic acid is an essential fatty acid that belongs to the omega-3 fatty acids group. It is reported to inhibit the synthesis of COX-2- and COX-1-catalyzed prostaglandin biosynthesis, resulting in reduced inflammation and the prevention of certain chronic diseases [29]. In addition, it was confirmed that other components with anti-inflammatory effects were included; the name and degree of inclusion of all chemicals are recorded in Table 1. Therefore, this also supports that Pp-EE has anti-inflammatory effects.

### 2.2. Inhibitory Effect of Pp-EE on Inflammatory Gene Expression and Transcription Factors

**Figure 2:**
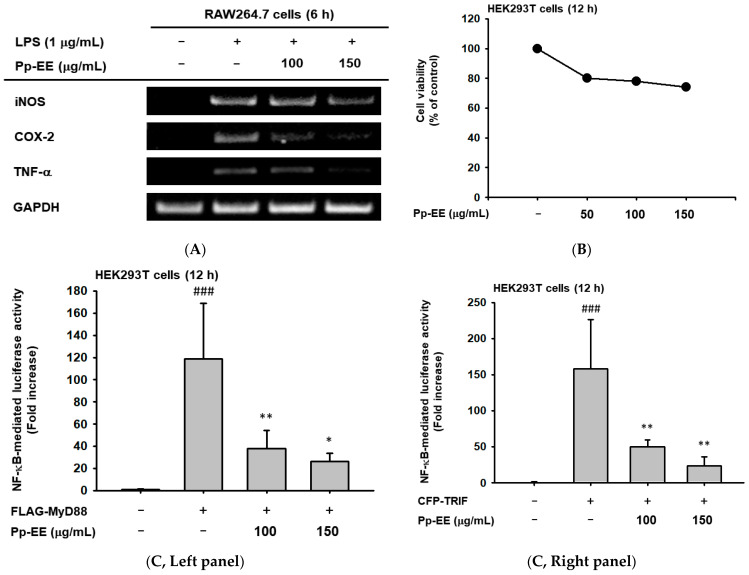

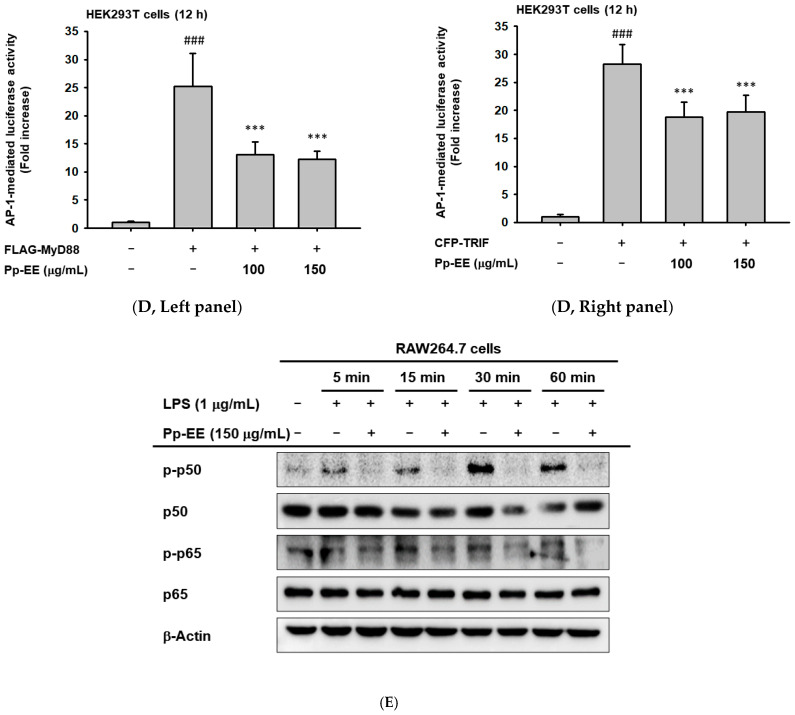
Pp-EE exerts influence on decreasing expressions of mRNA and transcription factor. (**A**) Changes in mRNA expression level in RAW264.7 cells induced stimulation through LPS after pretreatment of Pp-EE (0–150 μg/mL). (**B**) Cytotoxicity of Pp-EE for HEK293T cells with indicated concentrations of Pp-EE for 12 h. (**C**,**D**) The effect of Pp-EE (0–150 μg/mL) on the activation of transcription factor (NF-κB or AP-1). HEK293T cells were co-transfected with an NF-κB or AP-1 luciferase construct and β-gal plasmid for control with or without MyD88 (left panel) or TRIF (right panel). (**E**) The total and phosphorylated levels of p50, p65, and β-actin analyzed by immunoblotting assay. LPS (1 μg/mL)-stimulated RAW264.7 cells with or without Pg-EE (150 μg/mL) were prepared. ###: *p* < 0.001 compared to the normal group, *: *p* < 0.05, **: *p* < 0.01, and ***: *p* < 0.001 compared to the control group. −: no treatment and +: treatment.

Based on the previous experiment, we examined the changes in the mRNA expression levels of inflammatory cytokines. In this process, to confirm the anti-inflammatory effect, representative pro-inflammatory cytokines, including inducible nitric oxide synthase (iNOS), which is associated with a NO production reduction previously identified, were selected [30,31]. The results of the experiment showed that the transcription levels of iNOS, COX-2, and TNF-α were reduced in a concentration-dependent manner (Figure 2A).

Next, we conducted an experiment to confirm the change in activity of transcription factors regulating mRNA transcription by Pp-EE. Prior to the start of the experiment, we confirmed cytotoxicity in HEK293T cells used in a luciferase assay under the same conditions as before, and Pp-EE (0–150 μg/mL) also showed no cytotoxicity (Figure 2B). We performed a luciferase reporter gene assay to see how Pp-EE would affect the activation of transcription factors with induction on HEK293T cells via MyD88 or TRIF. Activation levels of NF-κB and AP-1 were high in the presence of TRIF and MyD88, which decreased in a concentration-dependent manner when Pp-EE was treated (Figure 2C,D). On the other hand, when comparing the two, the reduction effect was better in NF-κB than in AP-1. Therefore, in this paper, we try to confirm the efficacy by focusing on the anti-inflammatory effect on the NF-κB signaling pathway. 

After confirming that Pp-EE reduces the activity of inflammatory-related transcription factors, we tried to understand more specifically how it affects the signaling system that causes inflammation. In order to confirm the action on the NF-κB signaling pathway, it was first checked whether the activation of p50 and p65, the common dimers of NF-κB, had changed. We pretreated Pp-EE in RAW264.7 cells for 30 min and then increased the time to induce inflammatory reactions using LPS to 5, 15, 30, and 60 min to confirm the results using the cell lysate. As a result, it was confirmed that the phosphorylation of NF-κB subunits was decreased when Pp-EE was treated (Figure 2E).

### 2.3. Pp-EE Inhibits Phosphorylation of Src in the NFκB Signaling Pathway

**Figure 3:**
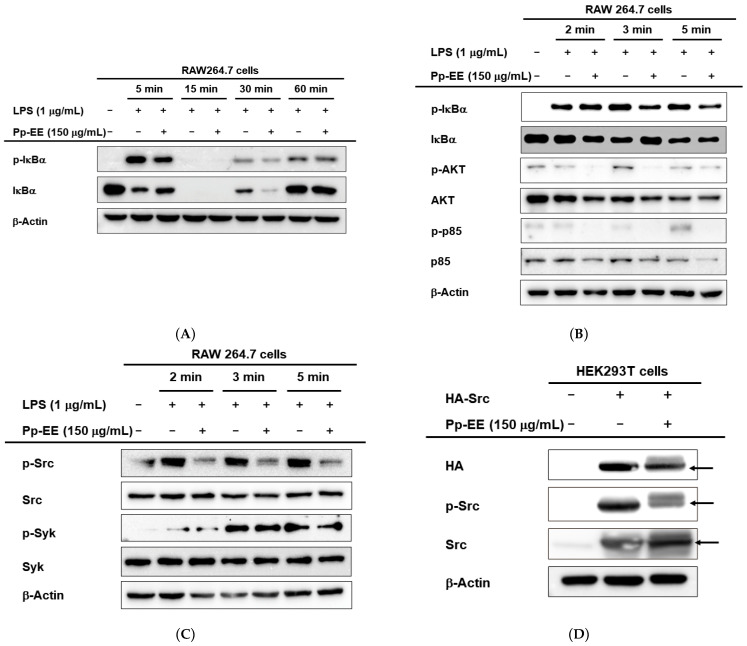

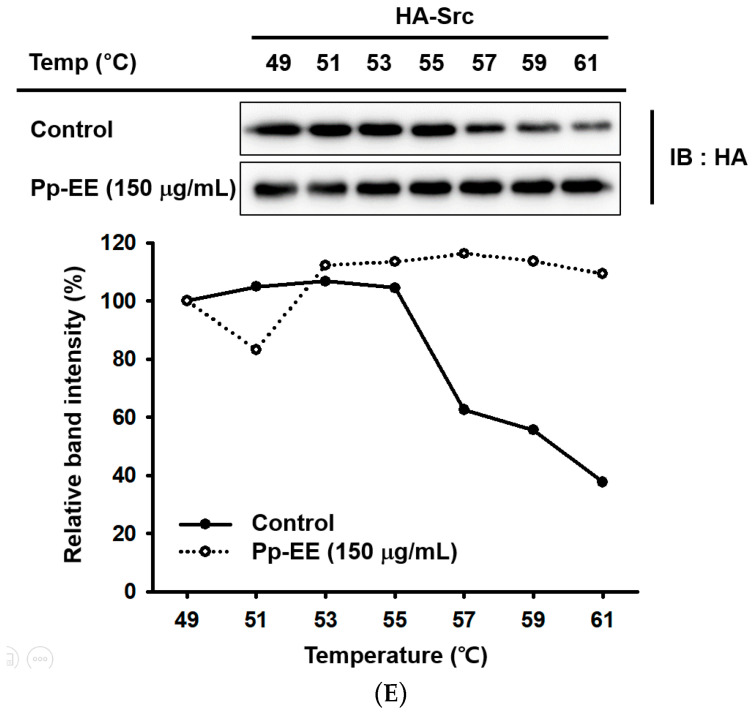
Pp-EE targeting Src of the NF-κB signal pathway. (**A**–**C**) RAW264.7 cells were pretreated with Pp-EE (150 µg/mL) for 30 min and induced an inflammatory response by LPS (1 μg/mL) at different times. Phosphorylation level and total level of IκBα, AKT, p85, Src, and Syk were detected by Western blot. (**D**) Verification in HEK293T cells with HA-Src over-expression. Total and phospho-forms of Src and β-actin were examined by Western blotting. (**E**) Stabilizing effects of Src protein through interaction with Pp-EE were confirmed by CETSA. Western blotting was performed on the cell lysate, and the calculation of band intensity of Src was carried out through Image J. −: no treatment and +: treatment.

To confirm the specific target of the signaling pathway, we continued to confirm the effect on the activation of other signaling molecules. Phosphorylation of inhibitor of kappa B alpha (IκBα), which binds to NF-κB in the cytoplasm to form an inactive complex, was confirmed under the same experimental conditions. Except for the disappearance of the IκBα protein at 15 min as a consequence of its proteolytic degradation [32], the activation of the IκBα protein was inhibited in the results after 30 min (Figure 3A).

Next, it was attempted to confirm whether Pp-EE exhibited its inhibitory effect for the activation of AKT and p85 (upper signal transmission substances) and IκBα, which were confirmed to have decreased activity. RAW264.7 cells were pretreated with Pp-EE for 30 min and then inducted through LPS for 2, 3, and 5 min. It was observed that the activity was decreased after 3 min for IκBα, and the activity was suppressed after 2 min for AKT and p85 (Figure 3B). Finally, experiments were conducted on Src and Syk, which are higher signal transmission substances, under the same conditions, and unlike Syk, which did not show a change, the activation degree of Src was decreased from the time it was inducted for 2 min (Figure 3C). Therefore, we speculated that Pp-EE had an anti-inflammatory effect by controlling the activity of Src in such a way as to inhibit it.

To confirm this hypothesis, we conducted an experiment on how Pp-EE affects activation in a situation where over-expression was caused by the transfection of the Src gene to HEK293T cells. We transfected HA-tagged Src to the cell and compared the change with normal cells with no treatment of Pp-EE. It was confirmed that the phosphorylation of Src decreased significantly when Pp-EE was treated (Figure 3D). In addition, we tried to confirm the association between Src and Pp-EE in a different way. We checked the change in the thermal stability of the Src protein by treatment of Pp-EE in a situation where over-expression was caused by the transfection of the Src gene to HEK293T cells. As a result of this experiment, when Pp-EE was treated, there was no difference in the Src protein expression level, unlike in the control group in which Src was degraded. Thus, it was confirmed that Pp-EE exhibited anti-inflammatory effects by interacting with Src as a target (Figure 3E).

### 2.4. Effects of Pp-EE on an HCl/EtOH-Induced Gastritis Model

**Figure 4:**
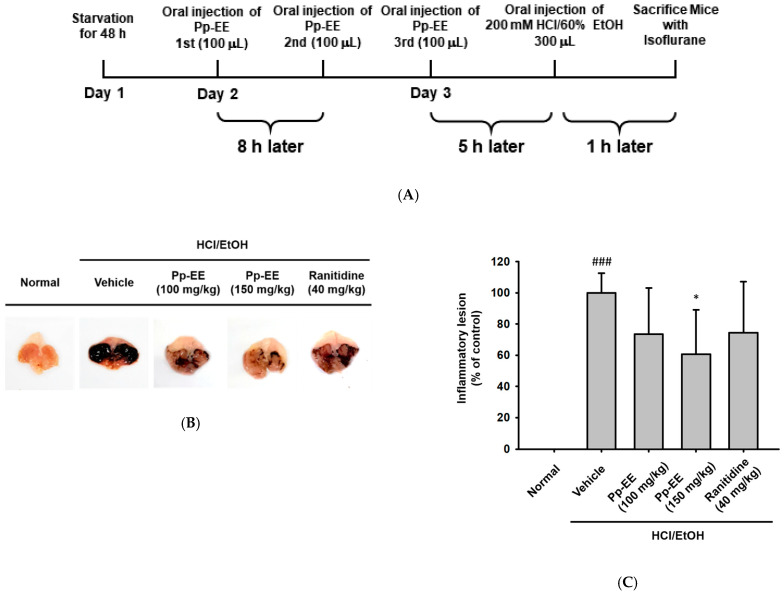

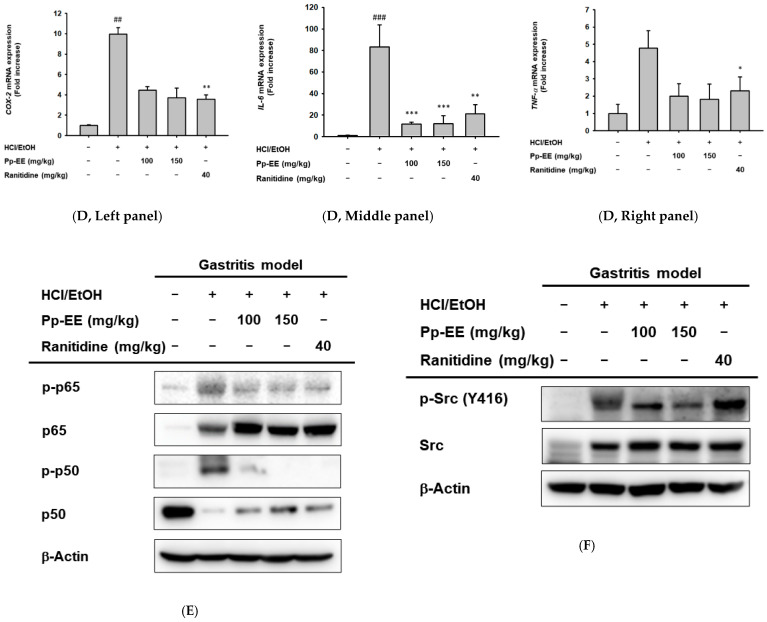
Anti-inflammatory effects on HCl/EtOH-induced gastritis model. (**A**) Schematic diagram of an experiment using the acute gastritis model. ICR mice were separated into a total of five groups and were orally administered three times 100 μL of the drug corresponding to each group: a normal group and a negative control group (0.5% carboxymethylcellulose (CMC)), treatment groups (100 mg/kg Pp-EE or 150 mg/kg Pp-EE), or positive control group (40 mg/kg ranitidine). Five hours after the last dose, mice were orally administrated 200 mM HCl/60% EtOH (300 μL/mouse) to induce acute gastritis and sacrificed after 1 h. (**B**,**C**) Dissected stomachs were photographed, and the incidence of lesions was quantified using ImageJ. (**D**) Comparison of mRNA expression of inflammatory genes (*COX-2* (Left panel), *IL-6* (Middle panel), and *TNF-α* (Right panel)) in the stomach was conducted using real-time PCR (**E**,**F**) The protein levels in the stomach were detected by an immunoblotting assay. ###: *p* < 0.001 and ##: *p* < 0.01 compared to the normal group, *: *p* < 0.05, **: *p* < 0.01, and ***: *p* < 0.001 compared to the control group. −: no treatment and +: treatment.

As previous results show, after completing the confirmation in vitro, we tried to determine whether Pp-EE actually exhibits anti-inflammatory efficacy even in animal models. We tried to confirm the effect using the gastritis model, one of the representative inflammatory models. Acute gastritis was induced by peroral injection of 200 mM HCl/60% EtOH after three doses of medications (0.5% CMC, 100 mg/kg Pp-EE, 150 mg/kg Pp-EE or 40 mg/kg ranitidine) for each group (Figure 4A). In both Pp-EE groups, the degree of lesion decreased, as in the positive control group (Figure 4B,C).

In addition, as previously confirmed, changes in mRNA expression level and protein phosphorylation related to the NF-κB pathway were identified from the extracted stomach after sacrifice. Pp-EE decreased the pro-inflammatory cytokine mRNA expression levels of *COX*-*2* (Left panel), *IL-6* (Middle panel), and *TNF-α* (Right panel) (Figure 4D) and the phosphorylation levels of p65, p50, and Src (Figure 4E,F). When all of these results are put together, we can form a conclusion that Pp-EE exerts anti-inflammatory effects, in particular by inhibiting the activation of Src, the key mediator in the NF-κB pathway.

## 3. Discussion

The inflammatory response, a defense mechanism to protect our body, goes through a complex and sophisticated process and affects various areas. Many experts recognize the importance of suppressing this major response, as abnormalities occur in the meantime, and excessive inflammatory reactions can lead to serious diseases. Therefore, various drugs with functions for suppressing inflammation have been developed. The development of medicinal plants is still a promising option as the development of new materials using natural products is recognized as a safe treatment. In fact, NSAID-based drugs, a typical anti-inflammatory drug used in classical practice, are generally known for their side effects [22]. Hence, many patients and medical staff are interested in treatment therapy with natural compounds; this was why we conducted this study.

Prior to starting the study, we performed screening tests on anti-inflammatory function against several natural product extracts (data not shown). The ethanol extract of *P. paradoxa* Nutt. had excellent effects. Therefore, we selected Pp-EE as the subject of study and tried to understand its anti-inflammatory efficacy in more detail. *P.* *paradoxa* Nutt. has been used as a medicinal plant in various regions since ancient times and many Cinquefoils (*Potentilla*) plants show anti-inflammatory effects [23,25,26,27]. For this reason, we conducted a study on *P. paradoxa* Nutt. for the development of natural material drugs. We attempted to clarify the specific mechanism of how Pp-EE acts at the molecular level through analysis in vitro and in vivo.

In conducting research, we initiated identification using macrophages that play an important role in inflammation, the representative immune response of innate immunity. Pp-EE efficiently inhibited NO production in the case where RAW264.7 cells were stimulated by LPS, Poly (I:C), or Pam3CSK4. These results suggested that Pp-EE showed anti-inflammatory efficacy for various TLR families, indicating the possibility that it could be developed as an effective drug; it did not show severe cytotoxicity even up to 150 μg/mL (Figure 1A,B). From these results, we can infer that Pp-EE shows broad anti-inflammatory effects with low cytotoxicity. These anti-inflammatory effects did not lag behind those of L-NAME, which was used as a positive drug (Figure 1C).

Additionally, it contains various kinds of phytochemicals that can contribute to suppressing the inflammatory response (Figure 1E). As a result of analyzing the components through GC/MS, it was found that the components contained large quantities of α-linolenic acid, *n*-hexadecoic acid, and 5-hydroxymethylfural, each of which has anti-inflammatory effects [33,34,35]. In addition, gamma-sitosterol, of which there is a high content, has an anti-diabetic effect, and it is likely that this extract may exhibit a complex beneficial effect [36]. Based on these analysis results, we were able to confirm that the anti-inflammatory effect exhibited by Pp-EE is caused by these phytochemicals.

In the experiment performed to check the differences in mRNA expression, pro-inflammatory cytokines (*iNOS*, *COX*-2, and *TNF*-α) were suppressed by Pp-EE in LPS-stimulated RAW264.7 cells (Figure 2A). The HEK293T cells used in the next luciferase assay were selected as they were useful for us to uniquely identify their function during the experiment because, unlike macrophages, they have no TLR4 receptor [37]. Pp-EE significantly affects both AP-1 and NF-κB pathways but significantly reduces its activation in the NF-κB pathway (Figure 2C,D). In addition, while showing this effect, it did not show cytotoxicity (Figure 2B). Therefore, for subunits p65 and p50 of NF-κB, the change in phosphorylation level was confirmed under different conditions of LPS induction time points, and it was confirmed that each phosphorylation was suppressed (Figure 2E). As a result, we were able to confirm that Pp-EE affects NF-κB.

Subsequently, we tried to specify the target molecule by checking the upper signal transmission material to grasp the mechanism in the pathway. We checked the activation of IκBα after 5, 15, 30, and 60 min from induction, confirming that the level significantly decreases with Pp-EE treatment (Figure 3A). After that, the degree of activation of the other signal molecules was confirmed according to the order in the signaling pathway, as we gradually shortened the induction time (Figure 3B). As the final step of confirmation, we assumed that Pp-EE directly affects Src and Syk, the top substances of the NF-κB signaling pathway. When the degree of phosphorylation of the two proteins was confirmed by reducing the induction condition to 2, 3, and 5 min, the activity of Src was suppressed at all time points (Figure 3C). Thereafter, in order to confirm Src as a target protein, we conducted an experiment by overexpressing Src in HEK293T cells (Figure 3C). By proceeding with the confirmation step-by-step in this way, it was possible to guess what the action of Pp-EE was through. The interaction of Pp-EE and the Src protein could be confirmed through the additional CETSA experiment (Figure 3D). In this way, through a series of experiments conducted in vitro, we confirm that it is the Src protein that is affected through interaction with Pp-EE.

Afterwards, we tried to determine whether these results were actually the same in vivo with animals (Figure 4). Pp-EE not only weakened the incidence of inflammatory lesions but also reduced the expression of pro-inflammatory cytokines and the activation of NF-κB-pathway-related proteins (Figure 4B–E). In other words, this Pp-EE proved its effectiveness by showing the same anti-inflammatory effect.

## 4. Materials and Methods

### 4.1. Materials

RAW264.7 cells and HEK293T cells were purchased from the American Type Culture Collection (ATCC) (Rockville, MD, USA). Roswell Park Memorial Institute (RPMI) 1640, Dulbecco’s modified Eagle’s medium (DMEM), and penicillin–streptomycin solution were purchased from Hyclone (Logan, UT, USA). Fetal bovine serum (FBS), Opti-MEM reduced serum medium and TRIzol were purchased from GIBCO (Grand Island, NY, USA).

LPS, L-NAME, ranitidine, Pam3CSK4, Poly(I:C), dimethyl sulfoxide (DMSO), (3-4,5-dimethylthiazol-2-yl)-2,5-diphenyl-tetrazolium bromide (MTT), polyethylenimine (PEI), sodium dodecyl sulfate (SDS), and O-nitrophenyl-beta-D-galactopyranoside (ONPG) were acquired from Sigma Chemical Co. (St. Louis, MO, USA). TRI reagent^®^ was acquired from Molecular Research Centre Incorporated (Cincinnati, OH, USA). Enhanced chemiluminescence (ECL) reagent was supplied by Amersham (Bath, UK). PCR primers specific for COX-2, iNOS, IL-6, TNF-α, and GAPDH were obtained from Macrogen (Seoul, Korea). Antibodies specific for phosphorylated and total forms of p65, p50, IκBα, p85, AKT, Src, Syk. human influenza haemagglutinin (HA), and β-actin were purchased from Cell Signaling Technology (Beverly, MA, USA).

### 4.2. Preparation of Pp-EE and Its Treatment

A 95% ethanol extract of Pp-EE was prepared with the arial part of *P. paradoxa* Nutt., as reported previously [38]. The arial part of *P. paradoxa* Nutt., purchased from Herbmaul (Seoul, Korea), was ground to a powder, which was then used for the extraction process. The extraction was performed with 127 g of the plant material and 890 mL of 95% ethanol for 2 h, three times. The extract was percolated through filter paper (3 mm; Whatman PLC, Kent, UK), condensed using a rotary evaporator (Büchi AG, Flawil, Switzerland), and lyophilized using a freeze dryer (Martin Christ Gefriertrocknungsanlagen, Osterode am Harz, Germany). Pp-EE powder was dissolved and used as a 100 mg/mL stock solution using DMSO as a solvent. The experiment was performed while using DMSO as vehicle control, with the same dilution level as the negative control. For the in vitro experiment, Pp-EE stock solution was dissolved in a culture medium. When making dilutions to treat cells, the dilution coefficient was considered so that the final concentration could be the target concentration we wanted. For the in vivo experiment, the powder was suspended in 0.5% sodium carboxymethylcellulose (0.5% CMC).

### 4.3. Cell Culture

RAW264.7 cells were cultured in RPMI1640 medium with 10% heat-inactivated FBS and 1% penicillin/streptomycin and subcultured once every two days. HEK293T cells were cultured in DMEM medium with 5% heat-inactivated FBS and 1% penicillin/streptomycin and subcultured once every three days. The cells were cultured in an incubator that maintained 5% CO_2_ and 37 °C.

### 4.4. Nitric Oxide (NO) Production Assay

RAW264.7 cells were plated in 96-well plates, with 100 μL per well at a density of 1 × 10^6^ cells/mL, and then incubated for about 16 h in an incubator. Pp-EE was diluted at 4 times the concentration of 50–150 μg/mL using the same medium used to grow cells (for sample groups, except for the highest concentration of Pp-EE, DMSO was added to each group to equalize the concentration conditions of the vehicle to the highest concentration) and 50 μL was administered to the compound group. For the normal group and the control group, DMSO was dissolved in the medium at the same concentration as the Pp-EE treatment group and administered in the same amount. After 30 min of pretreatment, an LPS stock solution (2 mg/mL) was diluted to 1 μg/mL in the medium, and 50 μL was administered to the control and compound groups except for the normal group. For the normal group, the same amount of medium was administered. After an induction of 24 h, 100 μL of the supernatant was moved to a new plate in the same arrangement, then the absorbance at 540 nm was measured after mixing in a 1:1 ratio with the Griess solution [39].

The same experiment was performed using Pam3CSK4 (10 μg/mL) or poly(I:C) (200 μg/mL) to induce the inflammatory response.

### 4.5. Cell Viability

We plated RAW264.7 cells in 96-well plates, with 100 μL at a density of 1 × 10^6^ cells/mL or HEK293T cells at a density of 2 × 10^5^ cells/mL, and incubated them for about 16 h. Afterwards, we treated the cells with specified concentrations (50–150 μg/mL) of Pp-EE or L-NAME (0–2 mM) or DMSO (for the normal group) dissolved in media. After an incubation of 24 h, 100 μL of the supernatant was discarded; then, cells were incubated with 10 μL of 5 mg/mL MTT solution in an incubator for about 3 h (enough time for formazan to be created) [18]. To dissolve the insoluble formazan crystals, 100 μL of MTT stopping solution was added and incubated overnight; then, the absorbance was detected at 570 nm.

### 4.6. Gas Chromatography–Mass Spectrometry (GC–MS/MS)

To analyze the phytochemical components of Pp-EE, GC–MS/MS was conducted, as reported previously [25]. Based on the type of analysis component and the integration value in the graph of each component, the amount of content compared to the total amount was identified, and the contents were summarized.

### 4.7. mRNA Expression Level Analysis by Semi-Quantitative Reverse Transcriptase–PCR and Quantitative Real-Time PCR

RAW264.7 cells were plated in 6-well plates, with 1 mL per well at a density of 1 × 10^6^ cells/mL, and then incubated for about 16 h in an incubator. After pretreating Pp-EE (0–150 μg/mL) for 30 min, LPS (1 μg/mL) was administered to the control and compound groups to induce an inflammatory reaction. After 6 h of induction, we discarded the medium and then extracted the total RNA using TRIzol reagent, following the manufacturer’s instructions. The mRNA was extracted from the ground gastrointestinal tissue in the same process above.

Using the mRNA, cDNA synthesis and reverse transcription PCR were performed in the same way as previously done [40]. cDNA was synthesized from 1 μg of total RNA using a RevertAid First Strand cDNA synthesis kit (Thermo Fisher Scientific, Waltham, MA, USA). 

In the case of mRNA extracted through cell experiments, it was synthesized through semi-quantitative RT-PCR and then electrophoresis on 1% agarose gel containing EtBr. The mRNA extracted from the mice’s stomachs was quantified using added SYBR green during real-time PCR [14]. The primers used for each experiment are presented in Table 2.

### 4.8. Plasmid Transfection and Luciferase Reporter Gene Activity Assay

HEK293T cells were plated in 24-well plates, with 400 μL per well at a density of 2.5 × 10^6^ cells/mL, and then incubated for about 16 h. Cells were co-transfected with MyD88 or TRIF vectors as the inducer, NF-κB-Luc or AP-1-Luc as the reporter, and β-gal plasmid as normalization. All vectors and PEI were diluted with Opti-MEM. When 24 h had elapsed from transfection, the cells were treated with Pp-EE (0–150 μg/mL) dissolved in the medium. When 24 h had passed since the treatment of Pp-EE, cells were lysed by adding luciferase lysis buffer and frozen at −70 °C for more than 3 h, then thawed. We collected the cell lysate in tubes and centrifuged them at 12,000 rpm for 1 min at 4 °C. Only the supernatant was then transferred to the new tube and used to measure the results. The luciferase reporter activity was measured by checking the luminescence after the luciferin and supernatant were mixed [18,41]. The activity of β-galactosidase was measured by reacting with ONPG and used to normalize transfection efficiency.

### 4.9. Whole Lysate Preparation and Western Blotting Assay

RAW264.7 cells were plated in 3 cm plates, with 1 mL at a density of 1 × 10^6^ cells/mL, and treated with Pp-EE for 30 min. After the specific time for treatment with LPS, all medium was suctioned, and then the cells were washed and collected in tubes using PBS.

Plated HEK293T cells at a density of 3 × 10^5^ cells/mL were transfected with HA-Src using PEI in the state of being dissolved in Opti-MEM for 24 h; then, the Pp-EE or DMSO dissolved in the medium was treated. Cells were collected in the same process as RAW264.7 cells. Each cell was centrifuged at 12,000 rpm for 1 min at 4 °C; then, the pellet was lysed using whole lysis buffer. For the composition of the whole lysis buffer, the previous experiment was referred to [40]. 

All samples were quantified at a concentration of 20 μg/mL and then separated by sodium dodecyl sulfate–polyacrylamide gel electrophoresis (Bio-Rad, Hercules, CA, USA) based on their molecular weight. After proteins in the gel were transferred to the membrane, immunoblotting was performed with specific antibodies, as reported previously [42,43]. With whole cell lysates from cell lines or animal tissue, the phosphorylated or total forms of p50, p65, IκBα, AKT, p85, Src, Syk, HA, and β-actin were measured.

### 4.10. Cellular Thermal Shift Assay (CETSA)

HEK293T cells were seeded into a 6-well plate, with 1 ml at a density of 1.5 × 10^5^ cells/mL, and transfected with Src using PEI. DMSO or Pp-EE was administered to the cells after 24 h from transfection; then, the cells were washed using PBS. We collected whole cells and calculated the cell density of the resuspension from each group; based on the lower density group, PBS was added to the higher density group to match the two equally. Then, the cells were divided equally into seven PCR tubes of 100 μL each. Using a thermocycler, the tubes were heated to a temperature range of 42 to 60 °C degrees, and a cooling process at 25 °C degrees was performed for 3 min, respectively. After that, the cells were lysed by freezing at −70 °C degrees for more than 3 h. Then, the cell lysates were thawed and centrifuged at 13,000 rpm for 30 min under the condition of 4 °C. The protein supernatant was then separated through gel electrophoresis and analyzed through Western blotting. The intensity of the bands was quantified using ImageJ software. The procedure of the experiment was conducted by referring to previous studies [27].

### 4.11. Animals

Institute of Cancer Research (ICR) mice (male, 5 weeks old, 17–21 g) were obtained from Orient Bio (Gyeonggi, Korea) and housed in plastic cages under a 12 h light–dark cycle. Water was given ad libitum, and feed (Samyang, Daejeon, Korea) was limited only on the first day of the experiment. All studies were conducted in agreement with the guidelines of the Institutional Animal Care and Use Committee at Sungkyunkwan University (Suwon, Korea; approval ID: SKKUIACUC2021-04-19-1).

### 4.12. EtOH/HCl-Induced Gastritis Mouse Model

ICR mice divided into groups (4 mice/group) fasted for 48 h to empty their stomachs, and on the second day, mice were given orally administrated 100 μL of Pp-EE (150 mg/kg or 100 mg/kg) or ranitidine (40 mg/kg) two times in 8 h intervals. On the third day, 5 h after the last dose, gastritis was induced with 200 mM HCl/60% EtOH (300 μL/mouse). After an hour, anesthesia and sacrifice were performed using isoflurane. The stomachs were harvested and dissected along the greater curvature. After opening the stomach, we washed it with PBS several times and photographed it. The photos were analyzed and compared with the degree of lesion occurrence using the Image J program, as reported previously [44,45]. The stomach tissue was ground with liquid nitrogen and stored at −70 °C until use.

### 4.13. Statistical Analysis

A Student’s *t*-test and one-way ANOVA were performed to determine the statistical significance between the experimental group, the control group, and the normal control group. Data are expressed as the standard error of the mean, and the results were obtained from at least three independent experiments performed in triplicate. A *p*-value <0.05 was considered statistically significant.

## 5. Conclusions

**Figure 5:**
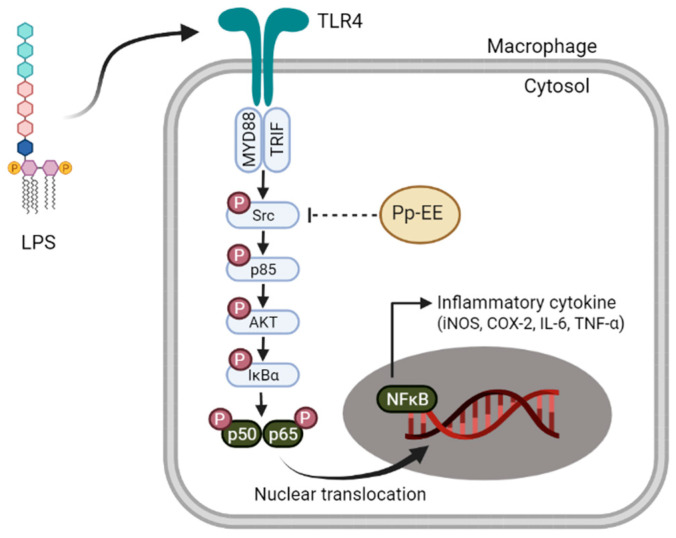
Schemes for mechanisms in which Pp-EE has anti-inflammatory efficacy targeting Src.

In this paper, we have conducted experiments on how Pp-EE has anti-inflammatory effects in vitro and in vivo. Pp-EE interacts with Src, the initial signaling agent of the NF-κB signaling pathway, inhibiting its activation, thereby inhibiting inflammation-induced reactions and exhibiting anti-inflammatory effects (Figure 5). These findings suggest the possibility of developing Pp-EE as a natural material drug that can control inflammatory diseases such as gastritis. However, in this study, it was not possible to confirm the other transcription factor that showed an effect, the AP-1 signaling pathway. Since immune cells other than macrophages were not used in identifying the function of controlling inflammation, it seems necessary to study the relevant matters. In addition to gastritis, we expected to understand the wider use of Pp-EE in future studies, including checking whether it is effective in animal models such as enteritis or pneumonia.

## Figures and Tables

**Table 1:** Phytochemical analysis of Pp-EE by GC/MS.

Peak No.	R.T. Min	Name of the Chemical	Corr. Area	% of Total
1	1.759	Acetic acid	21,109,327	1.635%
2	2.083	3-Isopropoxypropylamine	10,501,824	0.813%
3	3.254	Glyceraldehyde	10,182,555	0.789%
4	3.850	Dihydro-2(3H)-thiophenone	16,852,589	1.305%
5	4.026	Dihydroxyacetone	23,960,724	1.856%
6	5.219	1,3,4-Thiadiazol-2-amine	3,874,410	0.300%
7	6.599	1,3,5-Triazine-2,4,6-triamine	30,477,893	2.360%
8	7.617	2,3-Dihydro-3,5-dihydroxy-6-methyl-4-pyrone	34,525,448	2.674%
9	7.785	4-Hydroxydihydro-2(3H)-furanone	3,349,145	0.259%
10	8.629	2,3-Dihydrobenzofuran	10,411,652	0.806%
11	8.784	5-Hydroxymethylfurfural	55,030,241	4.262%
12	9.007	3-Hydroxypropane-1,2-diyl diacetate	30,797,862	2.385%
13	9.557	3-Hydroxy-2,3-dihydromaltol	55,222,588	4.277%
14	10.042	2-Methoxy-4-vinylphenol	11,629,996	0.901%
15	10.419	2,7-Oxepanedione	28,292,149	2.191%
16	10.892	1,2,3-Benzenetriol	15,699,148	1.216%
17	11.644	Glutaric acid, 2-fluorophenyl 3-nitrobenzyl ester	14,647,966	1.134%
18	11.840	Trans-2-Isobutyl-4-methyl-1,3-dioxolane	32,966,709	2.553%
19	14.007	Methyl alpha-d-ribopyranoside	237,519,046	18.395%
20	16.218	Neophytadiene	2,958,855	0.229%
21	16.583	Phthalic acid, 7-bromoheptyl isobutyl ester	5,313,241	0.411%
22	17.433	n-Hexadecanoic acid	93,083,412	7.209%
23	17.527	Dibutyl phthalate	15,955,128	1.236%
24	17.758	Hexadecanoic acid, ethyl ester	11,265,920	0.873%
25	18.910	Phytol	32,677,229	2.531%
26	19.164	9,12,15-Octadecatrienoic acid, (Z, Z, Z)-	211,802,731	16.404%
27	19.316	Octadecanoic acid	30,759,152	2.382%
28	19.419	9,12,15-Octadecatrienoic acid, ethyl ester, (Z, Z, Z)-	19,525,879	1.512%
29	21.100	9-Octadecenamide, (Z)-	11,897,164	0.921%
30	21.717	Kauren-19-oic acid	3,792,760	0.294%
31	22.272	Hexadecanoic acid, 2-hydroxy-1-(hydroxymethyl) ethyl ester	21,655,818	1.677%
32	22.610	1,2-Benzenedicarboxylic acid, bis (2-ethylhexyl) ester	10,049,832	0.778%
33	23.662	9,12-Octadecadienoic acid (Z, Z)-, 2-hydroxy-1-(hydroxymethyl) ethyl ester	9,191,159	0.712%
34	23.732	Linolenic acid, 2-hydroxy-1-(hydroxymethyl) ethyl ester (Z, Z, Z)-	31,457,746	2.436%
35	23.940	Thunbergol	10,918,879	0.846%
36	24.910	2-(Acetoxymethyl)-3-(methoxycarbonyl) biphenylene	4,408,873	0.341%
37	25.069	2-Ethylacridine	6,372,792	0.494%
38	27.168	Vitamin E	16,324,800	1.264%
39	28.301	Hexamethylcyclotrisiloxane	4,756,890	0.368%
40	29.386	gamma-Sitosterol	84,425,037	6.539%
41	30.595	2-tert-Butylphenol, tert-butyldimethylsilyl ether	5,550,719	0.430%

**Table 2:** Primer sequences used in analysis of mRNA expression levels of pro-inflammatory cytokine genes by semi-quantitative RT-PCR and quantitative real-time PCR.

Gene (Type)	Direction	Sequences (5′ to 3′)
COX-2 (semi-RT-PCR)	Forward	TCACGTGGAGTCCGCTTTAC
	Reverse	TTCGACAGGAAGGGGATGTT
COX-2 (real-time PCR)	Forward	TTGGAGGCGAAGTGGGTTTT
	Reverse	TGGCTGTTTTGGTAGGCTGT
iNOS (semi-RT-PCR)	Forward	TGCCAGGGTCACAACTTTACA
	Reverse	ACCCCAAGCAAGACTTGGAC
IL-6 (semi-RT-PCR)	Forward	GCCTTCTTGGGACTGATGG
	Reverse	TGGAAATTGGGGTAGGAAGGAC
IL-6 (real-time PCR)	Forward	AGCCAGAGTCCTTCAGAGAGA
	Reverse	AGGAGAGCATTGGAAATTGGGG
TNF-α (semi-RT-PCR)	Forward	TGCCTATGTCTCAGCCTCTT
	Reverse	GAGGCCATTTGGGAACTTCT
TNF-α (real-time PCR)	Forward	TTGACCTCAGCGCTGAGTTG
	Reverse	CCTGTAGCCCACGTCGTAGC
GAPDH (semi-RT-PCR)	Forward	GAAGGTCGGTGTGAACGGAT
	Reverse	AGTGATGGCATGGACTGTGG
GAPDH (real-time PCR)	Forward	TGTTGAACGGATTTGGCCGTA
	Reverse	ACTGTGCCGTTGAATTTGCC

## Data Availability

The data used to support the findings of this study are available from the corresponding author upon request.

## Abbreviations

AP-1	Activator protein-1
COX-2	Cyclooxygenase-2
HA	Haemagglutanin
iNOS	Inducible nitric oxide synthase
IκBα	Inhibitor of kappa B alpha
IL-1β	Interleukin-1β
IL-6	Interleukin 6
MyD88	Myeloid differentiation primary response 88
NO	Nitric oxide
p85	Phosphoinositide-3-kinase p85
Pp-EE	*Potentilla paradoxa* Nutt. Ethanol extract
LPS	Lipopolysaccharide
NF-κB	Nuclear factor-κB
TRIF	TIR-domain-containing adapter-inducing interferon-β
TNF-	Tumor necrosis factor-alphaα
